# Correlation between biofilm formation and resistance toward different commonly used antibiotics along with extended spectrum beta lactamase production in uropathogenic *Escherichia coli* isolated from the patients suspected of urinary tract infections visiting Shree Birendra Hospital, Chhauni, Kathmandu, Nepal

**DOI:** 10.1186/s13756-016-0104-9

**Published:** 2016-02-15

**Authors:** Sanjeev Neupane, Narayan Dutt Pant, Saroj Khatiwada, Raina Chaudhary, Megha Raj Banjara

**Affiliations:** Central Department of Microbiology, Tribhuvan University, Kirtipur Kathmandu, Nepal; Department of Microbiology, Grande International Hospital, Dhapasi Kathmandu, Nepal; Department of biochemistry, CIST College, Kathmandu, Nepal; Shree Birendra Hospital, Chhauni Kathmandu, Nepal

**Keywords:** *Escherichia coli*, Urinary tract infection, Extended spectrum beta lactamase, Biofilm, Nepal

## Abstract

**Background:**

*Escherichia coli* is the most predominant causative agent of urinary tract infection (UTI). Recently, increase in drug resistance among the uropathogenic bacteria has caused great problem in treatment of UTI. The main objective of this research is to determine the correlation between biofilm formation and resistance toward different commonly used antibiotics along with extended spectrum beta lactamase production in uropathogenic *Escherichia coli*.

**Methods:**

The urine samples collected from the patients suspected of urinary tract infections (visiting Shree Birendra Hospital, Chhauni, Kathmandu, Nepal between July to December 2013) were cultured in cystine lactose electrolyte deficient (CLED) agar by using semi quantitative culture technique. Extended spectrum beta lactamase (ESBL) production was detected by combined disc diffusion technique and biofilm formation was detected by Congo red agar method. Chi-square test was applied and *p*-value < 0.05 was considered statistically significant.

**Results:**

Out of 1480 urine samples, *E. Coli* was isolated from 208 (14.1 %) samples. Of total 69 (33.2 %) ESBL producing uropathogenic strains of *E. coli*, 20 (29 %) were strong biofilm producers, 22 (31.9 %) were moderate biofilm producers, 11 (15.9 %) were weak biofilm producers and 16 (23.2 %) were biofilm non producers. Whereas among 139 ESBL non producing *E. coli*, 22 (15.8 %) were strong biofilm producers, 20 (14.4 %) were moderate biofilm producers, 13 (9.4 %) were weak biofilm producers and 84 (60.4 %) were biofilm non producers. Among total 108 biofilm producing *E. coli*, maximum resistance was observed toward cephalexin followed by amoxicillin and highest susceptibility was seen toward amikacin.

**Conclusion:**

The ability of biofilm formation was found to be significantly higher in ESBL producing strains of *E. coli* than that in ESBL non producing strains (*p* < 0.05). There was higher resistance rate to antimicrobial agents among biofilm producing strains of *E. coli* than that in biofilm non producing strains. According to our antimicrobial susceptibility pattern for *E. coli*, to start preliminary treatment for UTI in Nepal, we recommend to use amikacin or nitrofurantoin. Further, for the treatment of the UTI, the antibiotics should be selected on the basis of the urine culture and sensitivity report.

## Background

Urinary tract infection (UTI) is one of the most common bacterial infections acquired both in the community and hospital settings, affecting all age groups [1, 2]. Worldwide, around 150 million cases of UTI are diagnosed each year [3] and *Escherichia coli* is identified as the most common cause of UTI, accounting for 80 to 85 % of the cases [4–6].

Recently the haphazard uses of antibiotics have resulted in the worldwide spread of antibiotic resistance among the bacteria causing a major problem [7]. The emergence and worldwide rapid increase in prevalence of extended spectrum beta-lactamase (ESBL) producing bacteria that are multidrug resistant, pose treatment problem resulting in high morbidity, high mortality, and increased health care costs [8]. Biofilm production is a mechanism exhibited by several microbes to survive in unfavorable conditions. The bacterial biofilm is a structured community of bacterial cells enclosed in polymeric matrix and adherent to a surface [9]. Biofilm producing uropathogenic bacteria may be responsible for many recurrent UTIs [10]. The bacteria enclosed in the biofilm are highly resistant to antibiotic treatment [9].

In this study we are investigating the incidence of the ESBL producing *E. coli* in causing UTI. Further we are determining the correlation between biofilm formation and drug resistance with commonly used antibiotics (for treatment of UTI) along with ESBL production in *E. coli* isolated from the urine samples of the patients suspected of urinary tract infections. This is the first this type of study conducted in Nepal.

## Methods

A cross sectional study was conducted among the patients suspected of urinary tract infections (having symptoms like burning micturation, frequent or intense urge to urinate, back pain or lower abdominal pain, fever or chills etc.) visiting Shree Birendra Hospital, Chhauni, Kathmandu, Nepal since May to October 2013.

Total 1480 mid stream urine samples collected from the patients (out patients and in patients) suspected of urinary tract infections were cultured by the semi-quantitative culture technique [11]. The patients having laboratory or radiological evidence of other infections as the cause of the symptoms, patients with urinary catheterization and those who already have received antibiotics were excluded from our study. The bacterial isolates from the urine samples were identified by using microbiological techniques as described in the Bergey’s manual which include morphological appearance of the colonies, staining reactions and biochemical properties. The antimicrobial susceptibility testing was done by Kirby-Bauer disk diffusion technique as recommended by clinical and laboratory standards institute (CLSI) [12].

### Detection of ESBL producers

**Fig. 1:**
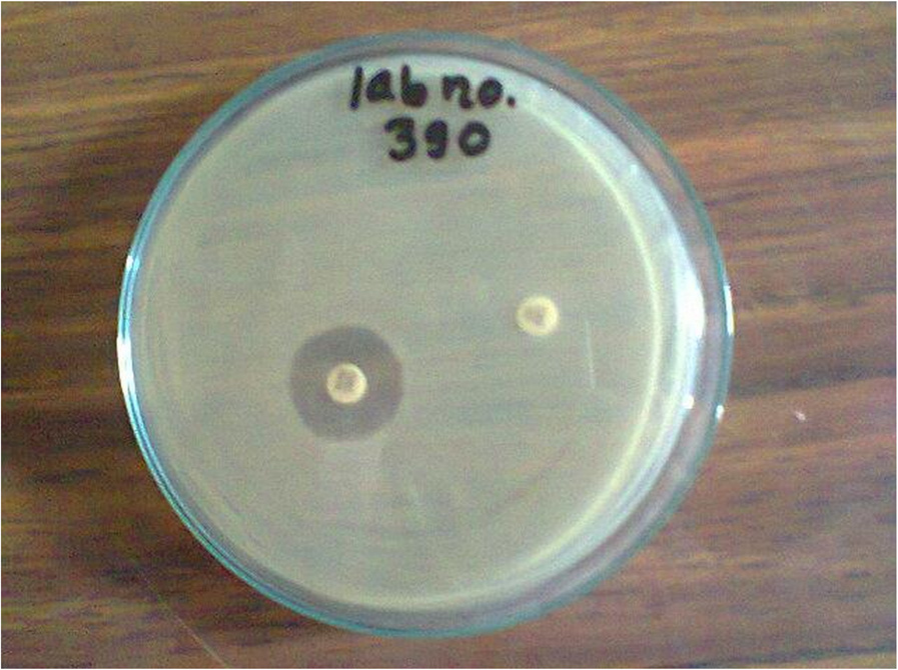
Confirmation of ESBL producer *E. coli* by combined disk method

Among the uropathogens isolated from suspected cases of UTI, only the strains of *E. coli* were subjected for detection of ESBL production. The phenotypic confirmation of the ESBL producing strains was done by combined disk method (Fig. 1) [12].

### Detection of biofilm producers

**Fig. 2:**
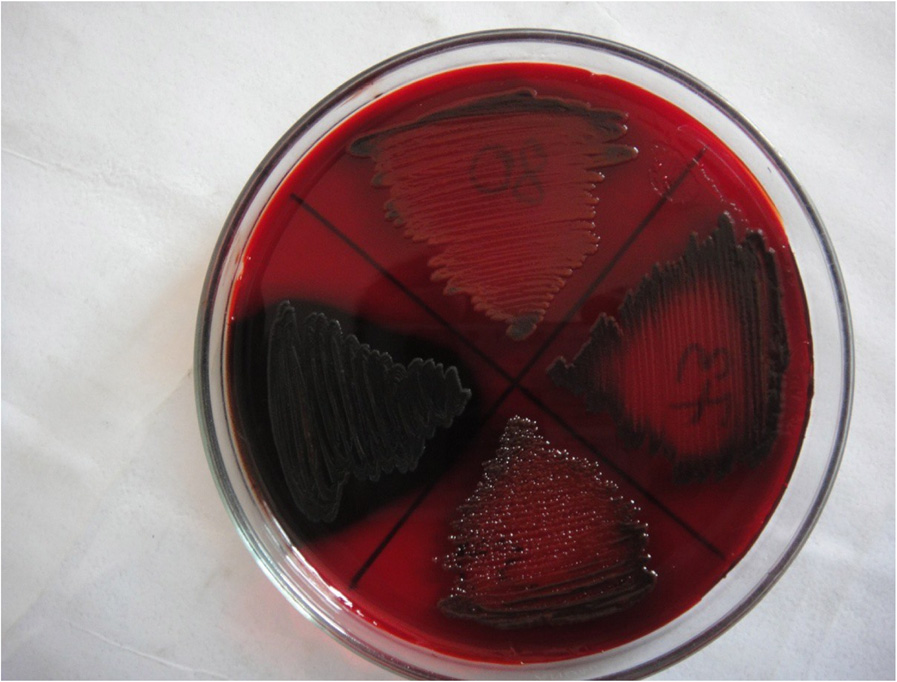
Biofilm detection test for *Escherechia coli* showing positive result on Congo red agar medium

Detection of biofilm production was done for both ESBL positive and ESBL negative strains of *E. coli* by Congo red agar method (CRA) (Fig. 2) [13].

On the basis of the intensity of color change of CRA medium after inoculation of the organisms, which is directly proportional to the amount of biofilm produced by the organisms, the biofilm producing organisms were classified into three categories as strong biofilm producers, moderate biofilm producers and weak biofilm producers [14, 15].

### Data analysis

SPSS version 16.0 statistical software package was used for statistical analysis. Chi-square test was applied. *P*-value < 0.05 was considered statistically significant.

## Results

Out of 1480 mid stream urine samples 278, (18.8 %) samples showed significant growth (≥ 10^5^ cfu/ml). *E. coli* was isolated from 208 (74.82 %) samples. Out of 208 *E. coli* isolates 69 (33.2 %) were found to be ESBL producers and 139 (66.8 %) were ESBL non producers. Among 208 *E. coli* isolates 42 (20.192 %) were found to be strong biofilm producers, 42 (20.192 %) were moderate biofilm producers, 24 (11.538 %) were weak biofilm producers and 100 (48.076 %) were biofilm non producers.

### Antibiotic resistance pattern of *E. coli* among biofilm producers and biofilm non producers

The antibiotic resistance among biofilm producing *E. coli* was found significantly higher than that of biofilm non producing *E. coli* (*p* < 0.05)*.* The correlation between biofilm production and antibiotic resistance was found statistically significant (*p* < 0.05) in most of the antibiotics (ciprofloxacin, ofloxacin, norfloxacin, amikacin, gentamicin, cotrimoxazole, cephalexin, cefixime, ceftazidime, cefotaxime, ceftriaxone and cefepime) but the correlation was not found to be significant in case of amoxicillin and nitrofurantoin (Table 1).

**Table 1:** Antibiotic resistance pattern of *E. coli* among biofilm producers and non producers along with the antibiotic susceptibility pattern of all the *E. coli* isolates

Antibiotics used	Resistance pattern of biofilm producers and biofilm non producers				Total Resistant	Total susceptible
	Strong producers *N* = 42	Moderate producers *N* = 42	Weak producers *N* = 24	Non producers *N* = 100		
	No (%)	No (%)	No (%)	No (%)	No (%)	No (%)
Ciprofloxacin	32 (76.2)	32 (76.2)	17 (70.8)	45 (45.0)	126 (60.6)	82 (39.4)
Ofloxacin	30 (71.4)	30 (71.4)	14 (58.3)	39 (39.0)	113 (54.3)	95 (45.7)
Norfloxacin	33 (78.6)	32 (76,2)	16 (66.7)	45 (45.5)	126 (60.6)	82 (39.4)
Gentamicin	24 (57.1)	26 (61.9)	6 (25.0)	26 (26.0)	82 (39.4)	126 (60.6)
Amikacin	7 (16.7)	11 (26.2)	3 (12.5)	5 (5.0)	26 (12.5)	182 (87.5)
Cotrimoxazole	27 (64.3)	29 (69,0)	17 (70.8)	42 (42.0)	115 (55.3)	93 (44.7)
Amoxicillin	40 (95.2)	37 (88.1)	22 (91.7)	87 (87.0)	186 (89.4)	22 (10.6)
Cephalexin	41 (97.6)	38 (90.5)	22 (91.7)	74 (74.0)	175 (84.1)	33 (15.9)
Cefixime	37 (88.1)	41 (97.6)	20 (83.3)	59 (59.0)	157 (75.5)	51 (24.5)
Ceftazidime	31 (73.8)	36 (85.7)	15 (62.7)	40 (40.0)	122 (58.7)	86 (41.3)
Cefotaxime	34 (81.1)	34 (81.0)	14 (58.3)	35 (35.0)	117 (56.2)	91 (43.8)
Ceftriaxone	29 (69.0)	28 (66.7)	15 (62.5)	28 (28.0)	100 (48.1)	108 (51.9)
Cefepime	31 (73.8)	27 (64.3)	12 (50.0)	33 (33.0)	103 (49.5)	105 (50.5)
Nitrofurantoin	12 (28.6)	11 (26.2)	6 (26.0)	28 (28.0)	57 (27.4)	151 (72.6)

### Antibiotic susceptibility pattern of the uropathogenic *E. coli* isolated

Of total 208 *E. coli* isolates, the highest numbers of the strains were susceptible to amikacin followed by nitrofurantoin, gentamicin, ceftriaxone and cefepime. Similarly least numbers of the strains were susceptible to amoxicillin (Table 1).

### Association of ESBL production and biofilm formation among *E. coli* isolates

Out of 69 ESBL producing uropathogenic strains of *E. coli*, 20 (29 %) were strong biofilm producers, 22 (31.9 %) were moderate biofilm producers, 11 (15.9 %) were weak biofilm producers and 16 (23.2 %) were biofilm non producers. Whereas among 139 ESBL non producing *E. coli*, 22 (15.8 %) were strong biofilm producers, 20 (14.4 %) were moderate biofilm producers, 13 (9.4 %) were weak biofilm producers and 84 (60.4 %) were biofilm non producers. The ability of biofilm formation was found to be significantly higher in ESBL producing strains of *E. coli* than that in ESBL non producing strains (*p* < 0.05).

## Discussion

Among 208 *E. coli* isolates, 108 (51.92 %) were biofilm producers. This finding agrees with the findings of different authors from different parts of the world [16, 17]. Biofilm protects bacteria from host defense mechanisms, along with the antibiotics [18].

In this study, the antibiotic resistance of biofilm producing *E. coli* was found significantly higher than that of biofilm non producing *E. coli* (*p* < 0.05)*.* Among biofilm producing *E. coli*, higher antibiotic resistance was observed in strong and moderate biofilm producers. The association between biofilm production and antibiotic resistance was found to be statistically significant (*p* < 0.05) except in case of amoxicillin and nitrofurantoin. Microorganisms growing in a biofilm are intrinsically resistant to many antibiotics increasing the antibiotic resistance up to 1000 folds and high antimicrobial concentrations are required to inactivate organisms growing in a biofilm [19, 20]. This may be because of the insufficient concentration of the antibiotics reaching some areas of the biofilms and metabolic inactiveness (along with the presence of active antibiotic degradation mechanisms contributing to halt the accumulation of the drugs up to an effective concentration) of the bacteria located at the base of the biofilms [9].

The biofilm forming ability was found to be significantly higher in ESBL positive strains of uropathogenic *E. coli* than that of ESBL negative strains [*p* < 0.05]. The study by Subramanian et al. in India also reported the higher ability of the ESBL producing organisms to form biofilm in comparison to that of ESBL non-producing isolates. It has been postulated that during occurrence of the large numbers of the chromosomal gene rearrangements upon acquisition of the ESBL plasmids the bacteria express several virulence genes [21].

ESBLs are enzymes that are responsible for resistance of bacteria toward third generation cephalosporins and monobactams [22]. Most of the plasmids responsible for ESBL production carry genes encoding resistance to other drugs also [23]. Due to frequent presence of cross-resistance to several other classes of antibiotics (like aminoglycosides and fluoroquinolones), in ESBL-producing organisms, the treatment of the infections by these bacteria are often present as the therapeutic challenges [22]. Further higher ability of the ESBL producing organisms to form biofilm makes the treatment even more difficult, increasing the mortality and severity of the infections [21]. Macrolides (erythromycin, clarithromycin, and azithromycin) are known to have antibiofilm activity against biofilm producing organisms by inhibiting a key component of the biofilm, alginate. And several studies have recommended, the combined therapy (being macrolides one of the first antibiotics chosen) as the treatment of choice in infections caused by biofilm producing organisms [9].

Increasing irrational and haphazard use of antibiotics, sales of substandard antibiotics and transmission of drug resistant bacteria among people may be responsible for the rise in antibiotic resistance among the bacteria [24]. Antimicrobial resistance has become a serious global public health issue. Infections caused by drug resistant bacteria are responsible for increased morbidity and mortality [25]. The selection of the antibiotics for treatment of the bacterial infections should be based on culture and sensitivity reports.

## Conclusion

The ability of biofilm formation was found higher among ESBL producing strains of *E. coli*. There was higher resistance rate among biofilm producing *E. coli* isolates to almost all the antimicrobial agents except a few. According to our antimicrobial susceptibility pattern for *E. coli*, to start preliminary treatment for UTI in Nepal, we recommend to use amikacin or nitrofurantoin. Further, for the treatment of the UTI, the antibiotics should be selected on the basis of the urine culture and sensitivity report.

### Limitations of the study

Due to lack of easy availability of the advanced laboratory in Nepal and due to lack of the fund we could not confirm the ESBL producing and biofilm producing organisms by using molecular technology.

## Abbreviations

ATCC: American type culture collection
CLED: Cystine lactose electrolyte deficient agar
CLSI: Clinical and laboratory standards institute
CRA: Congo red agar
ESBL: Extended spectrum beta lactamase
MHA: Mueller Hinton agar
SPSS: Statistical package for the social sciences
UTI: Urinary tract infection
